# Unusual presentation of dermatofibrosarcoma protuberans in a male patient’s breast: a case report and review of the literature

**DOI:** 10.1186/s12957-015-0562-1

**Published:** 2015-04-22

**Authors:** Mohannad Al Tarakji, Adriana Toro, Isidoro Di Carlo, Kulsoon Junejo

**Affiliations:** Department of Surgery, Hamad General Hospital, Al Rayyan Rd, 3050 Doha, Qatar; Department of Surgery, Taormina Hospital, Contrada Sirina, 98039 Messina, Italy; Department of Surgical Sciences, Organ Transplantation and Advanced Technologies, University of Catania, Via S. Sofia 78, 95100 Catania, Italy

**Keywords:** Dermatofibrosarcoma protuberans, Breast, Male

## Abstract

We herein present a case report and literature review of dermatofibrosarcoma protuberans in the breast of a male patient. A 27-year-old man presented with a painless lump in his right breast with areas of bluish skin discoloration. The diagnostic work-up comprised clinical examination, ultrasonography, core biopsy, mammography, and magnetic resonance imaging. After surgical excision, the preoperative diagnosis of dermatofibrosarcoma protuberans was proven by pathological examination and immunohistochemistry. The patient was still free of recurrence 1 year after surgical excision. This extremely rare case is, to the best of our knowledge, the fifth such case reported in the literature.

## Review

### Background

Dermatofibrosarcoma protuberans (DFSP) is a rare soft tissue neoplasm that was identified for the first time by Taylor in 1890 [1]. It was described in 1924 by Darier and Ferrand [2] as a progressive recurrent dermatofibroma and was later termed dermatofibrosarcoma protuberans by Hoffmann in 1925 [3].

DFSP is a rare soft tissue sarcoma that represents less than 5% of all soft tissue sarcomas occurring in adults aged 30 to 40 years [1]. The incidence of DFSP is five cases in every one million persons per year [4]. The most common sites are the trunk and extremities [5]. DFSP of the breast is extremely rare, especially in men. We herein provide a case report and literature review of DFSP in a male patient.

### Case presentation

A previously healthy 27-year-old Pakistani man presented with a painless lump in his right breast that was first noticed about 1.5 years previously. He initially noticed a thickened patch of skin, which gradually increased in size with areas of bluish discoloration. During the most recent couple of months, however, this patch of skin had become a lumpy swelling. The patient had no family history of breast, prostate, or testicular cancer. He had no systemic signs or associated weight loss.

**Figure 1:**
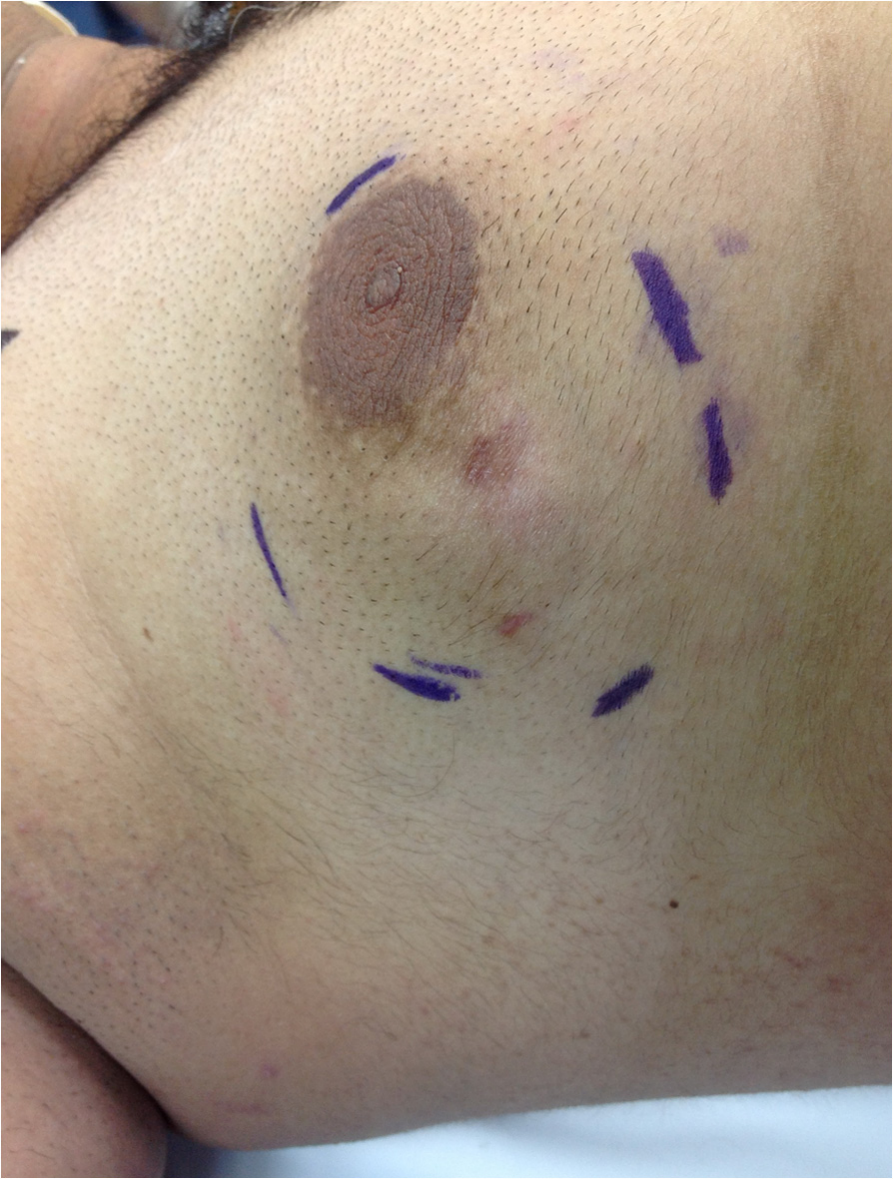
The skin over the tumor contained telangiectatic patches.

The patient’s general physical examination was unremarkable with the exception of his right breast, which contained a 4 × 3 cm lump in the lower outer quadrant that extended upward to the nipple. The lump was attached to the overlying skin but freely mobile on the underlying muscle. The skin over the tumor contained telangiectatic patches (Figure 1). There were no palpable axillary lymph nodes bilaterally.

**Figure 2:**
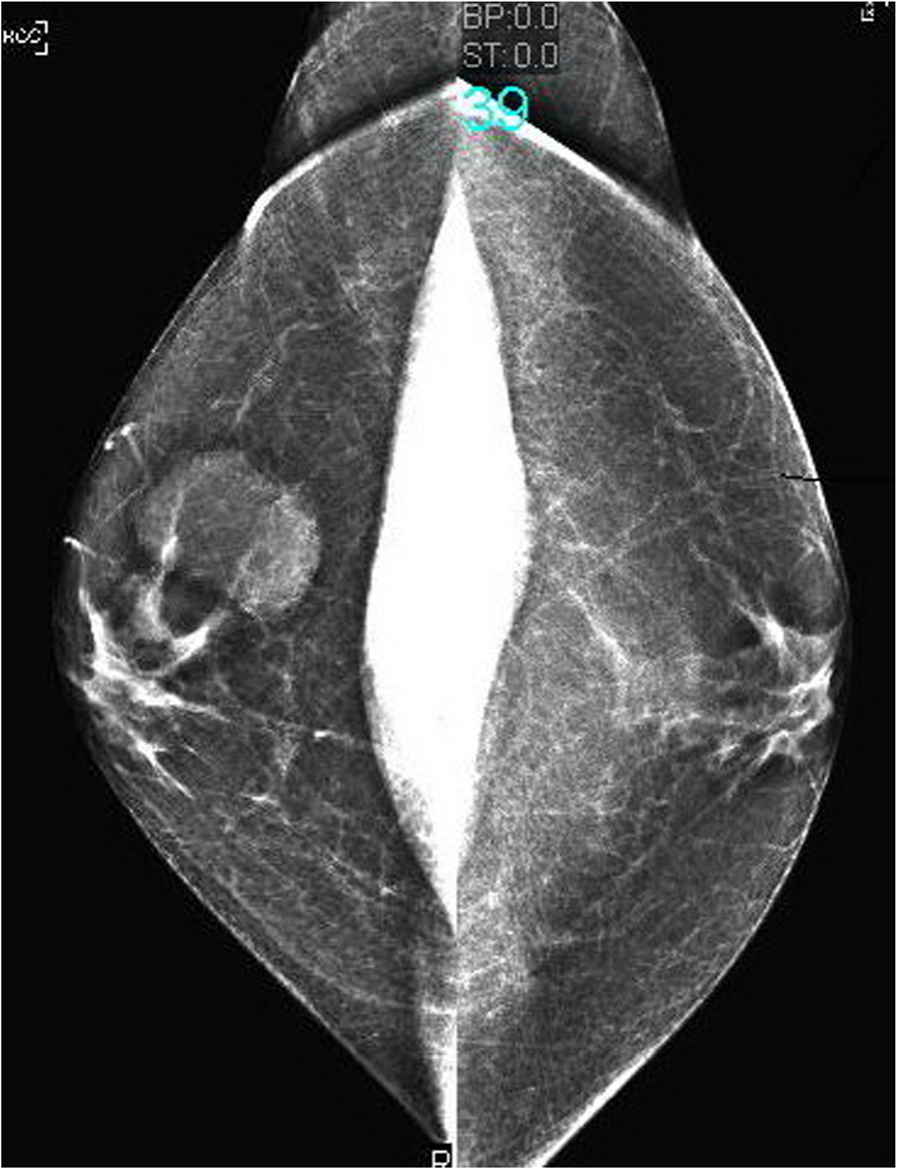
Mammography showed a mass in the right breast. The left breast was normal.

**Figure 3:**
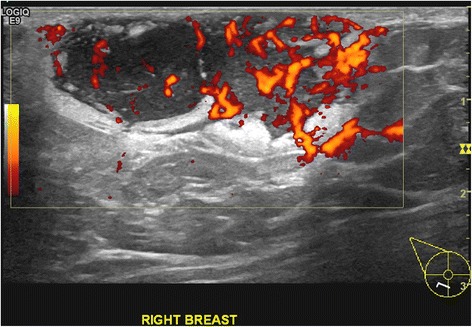
Ultrasound with power Doppler showed high vascularity of the mass.

**Figure 4:**
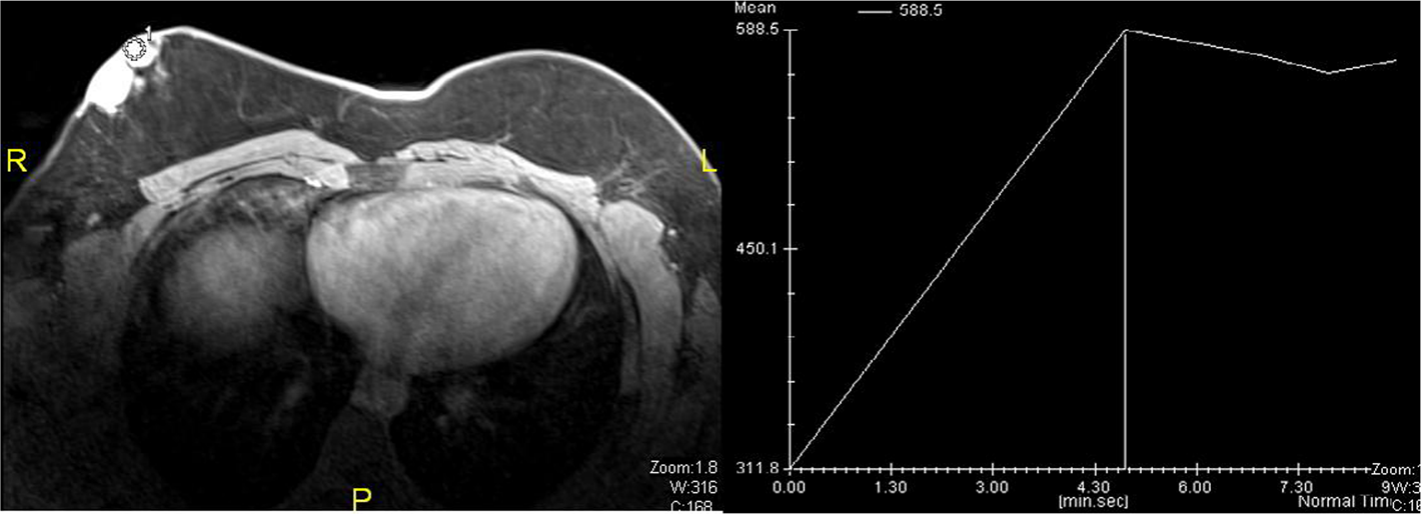
Magnetic resonance imaging showed intense enhancement of intravenous contrast with dye accumulation and washout in the right breast.

Mammography and ultrasonography of the breast showed a well-defined 35 × 27 mm hypoechoic lesion at the 7 to 8 o’clock position in the right breast. The lesion corresponded to the palpable nodule and showed increased vascularity on ultrasound (Figures 2 and 3). Magnetic resonance imaging (MRI) of the breast showed a lobulated 4.9 × 3.5 × 2.5 cm lesion with more intense signals on T1- and T2-weighted images than the muscle tissue. A plateau was present on the time-intensity curve. The lesion was graded as a Breast Imaging Reporting and Data System (BI-RADS) IV lesion, but malignancy could not be excluded (Figure 4). The left breast appeared normal. Bilateral normal axillary lymph nodes were present.

The patient underwent ultrasound-guided core biopsy of the lesion. Histopathological examination showed marked cellular proliferation comprising monomorphic spindle cells arranged into a tight storiform pattern (Figure 4). Immunohistochemically, the cells were positive for CD34, bc12, CD99, β-catenin, and vimentin. This morphology was very suggestive of dermatofibrosarcoma. Staging computed tomography and a bone scan showed no evidence of visceral or bone metastasis.

**Figure 5:**
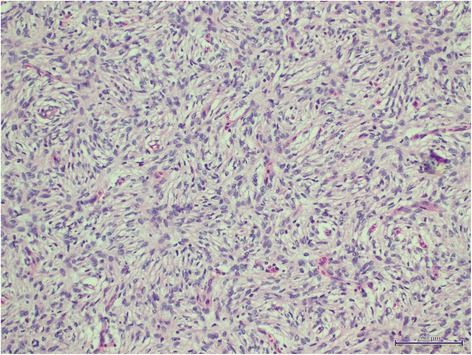
Astoriform spindle cell pattern was shown with hematoxylin and eosin staining, 200 ×.

The patient underwent wide local excision including the nipple-areolar complex. The pectoralis major fascia was excised along with the tumor. His postoperative recovery was uneventful. The final histopathological analysis revealed DFSD measuring 4.5 × 4.0 × 2.5 cm (Figure 5), with adequate surgical margins of 2.5 to 3.0 cm. No evidence of neoplasia was seen in the marginal skin or underlying fibroadipose tissue. The patient was still free of disease 12 months after the procedure.

PubMed and Google Scholar were used to search similar cases published in the literature. A search of PubMed from 1990 to 2014 revealed 69 articles on DFSP. Forty-one of these articles concerned DFSP of women’s breasts, 24 concerned patients unaffected by DFSP, and 2 concerned patients with DFSP not located in the breast. Of all 69 articles, only 2 were case reports of DFSP in a man’s breast. Reports of two additional male patients with DFSP of the breast were found in a search of Google Scholar during the same time period (Table 1) [6-9].

**Table 1:** **Literature search**

**Authors**	**Years**	**Age**	**Sex**	**Size (cm)**	**Side**
Chen [7]	2009	41	Male	4.5	Right
Park [8]	2011	36	Male	--	Right
Akhtar [6]	2012	22	Male	5	Left
Prabhu [9]	2014	55	Male	5	Left
Present case	2014	27	Male	4	Right

### Discussion

Primary breast sarcomas are rare mesenchymal tumors accounting for 0.2% to 1.0% of all breast malignancies [10] and less than 5% of all soft tissue sarcomas [11]. DFSP has an annual incidence of only 0.8 cases per million people and typically presents in mid-adult life with female predominance. DFSP may develop in patients of all ages, but those aged 30 to 40 years are primarily affected [1].

The most common sites of development of DFSP are the trunk (42% to 72%) and extremities (16% to 30%) [12]. However, Prabhu *et al*. reported rare sites including the hand, gluteal region, head and neck region, toe, vulva, and parotid gland. Localization in the breast is very rare [9].

DFSP is a soft tissue tumor of low-degree malignancy that arises in the dermis and spreads into the subcutaneous tissues and muscle [13]. DFSP has a low malignant potential, representing more a slow growing, localized ‘benign’ entity, but in 5% of the cases, this only nominally sarcoma undergoes sarcomatous transformation, and from that point often, after serial insufficient surgical excisions and recurrences, DFSP transforms to real aggressive sarcoma, often presenting with pulmonary metastases and even causing death of the patient.

Histologically, it is distinctively composed of monomorphic spindle cells arranged in a storiform pattern. Positive CD34 staining helps in the diagnosis [14]. Clinically, the lesion presents as a firm area of the skin, most commonly about 1 to 5 cm in diameter, with pink or violet-red plaques; the surrounding skin may be telangiectatic [15]. The tumors are generally fixed to the dermis but move freely over the more deeply lying tissue. However, they are often fixed to more deeply seated structures in advanced and/or recurrent cases [16]. DFSP tends to exhibit an indolent growth pattern and is usually less than 5 cm in size [17].

The morphology of the tumor requires a 3- to 4-cm surgical margin, and so, in most of the cases, a plastic surgical reconstruction is mandatory. The reason for the insufficient primary surgical care is often the fear from technical wound closure not involving the reconstructive plastic surgeon in the process.

Ultrasound exploration reveals an ovoid lesion with a circumscribed or microlobulated margin in the dermis or subcutaneous tissue, and the use of Doppler shows hypervascularization of the affected area [16]. Mammography reveals a dense lesion without fat or calcification [18]. Magnetic resonance imaging may be helpful to define the depth of infiltration of the tumor [18]. Fine needle aspiration cytology has been recommended as the first-line evaluation technique for superficial soft tissue tumors [19].

According to the NCCN Clinical Practice Guidelines in Oncology, the gold standard treatment is complete surgical excision with appropriate reconstruction [20]. Surgical excision with a margin of at least 2 to 3 cm is recommended for treatment because the local recurrence rate is 20% to 50% in cases of incomplete resection [4]. An unresectable lesion or resected lesion with a positive margin should be treated with adjuvant radiotherapy. Adjuvant radiotherapy can reliably reduce the local recurrence rate and avoid the mutilation and functional deficits caused by repeated surgery [21]. Chemotherapy in the form of imatinib has also been tried with encouraging results. Similar radiotherapy was attempted in a limited series, but the results did not favor its regular use [9].

Long-term follow-up requires strict ultrasonographic monitoring every 6 to 12 months with biopsy in cases of suspected recurrence. The 5-year survival rate of patients with DFSP is higher than 99% [22].

Our literature search revealed only four other cases of DFSP in the breast of a man. The mean age of these patients was 36.2 years, and the mean tumor size was 4.6 cm. To the best of our knowledge, the present case is the fifth.

## Conclusions

DFSP is a soft tissue sarcoma that very rarely develops in the breast of male patients. The clinical presentation includes a painless, gradually enlarging nodule with a thickened patch of skin exhibiting telangiectasia. The diagnosis is based on ultrasonography, mammography, MRI, and fine needle aspiration cytology. Surgical excision with wide margins may reduce the risk of recurrence.

### Consent

Written informed consent was obtained from the patient for publication of this case report and any accompanying images. A copy of the written consent is available for review by the Editor-in-Chief of this journal.
